# Self-perception of competences in clinical practice among recently graduated physicians from Lima, Peru

**DOI:** 10.1016/j.heliyon.2020.e05424

**Published:** 2020-11-16

**Authors:** Wendy Nieto-Gutierrez, Jessica Hanae Zafra-Tanaka, Kevin Pacheco-Barrios, Alvaro Taype-Rondan

**Affiliations:** a Facultad de Ciencias de la Salud, Universidad Científica del Sur, Lima, Perú, Panamericana Sur km 19. Lima 42, Perú; b CRONICAS Center of Excellence in Chronic Diseases, Universidad Peruana Cayetano Heredia, Lima, Peru; c Unidad de Investigación para la Generación y Síntesis de Evidencias en Salud, Universidad San Ignacio de Loyola, Lima, Peru

**Keywords:** Psychology, Medical education, Medical education undergraduate, Professional competence

## Abstract

**Objective:**

To describe the self-perception of basic competencies in clinical practice and evaluate their associated factors, among recently graduated physicians from Lima, Peru.

**Methods:**

Cross-sectional study. We evaluated the self-perception of the competencies in recently graduated physicians of four dimensions of the Tuning Project. Each item had six possible responses on a Likert scale: “non-existent” (1 point), “insufficient” (2 points), “sufficient” (3 points), “good” (4 points), “very good” (5 points) and “excellent” (6 points). To evaluate associated factors of the average scores for each dimension, we used linear regressions with the bootstrap method.

**Results:**

We analyzed data from 425 (54.9% were between 22 and 25 years old), which represent 31.1% of all physicians who graduated in 2016 from all medical schools located in Lima. The average self-perception score of the assessed dimensions was, in descending order: 4.49 for carrying out a patient consultation with a patient; 4.13 for carrying out practical procedures; 4.12 for providing immediate care of medical emergencies; and 4.04. for applying the principles, skills, and knowledge of evidence-based medicine (EBM). Regarding the factors associated with the average score per dimension, physicians from one university had higher average scores in all dimensions, and having done an externship and done an internship at social security hospitals was associated with a higer score with self-perception in two dimensions.

**Conclusion:**

Self-perception of competence was greater for the patient consultation dimension, and lower for the EBM. Only physicians from one university had higher average scores in all dimensions.

## 1 Introduction

Medical training should ensure the acquisition of the basic competencies a physician will need to achieve proper job performance [1, 2]. Although there is no consensus on what such competencies should be, some initiatives have been proposed, such as the “Tuning Project” [3, 4]. The Tuning Project which aims to establish the minimum general competencies a physician must have was launched by the European Commission [2, 5, 6], and has also been contextualized for Latin America with the participation of different universities in the region, including some universities in Peru [4, 7].

Previous studies conducted in Portugal [8], UK [9, 10, 11], Zambia [12], China [13], Chile [14, 15], and Mexico [16] have evaluated the competencies acquired by medical students during their undergraduate training, reporting heterogeneous methodology and results. However, we have not found studies conducted in Peru, a country with particular needs concerning medical education.

In Peru, most of the recently graduated physician enrol for the Rural Health Service (SERUMS, by its acronym in Spanish), a social service that lasts a year, in which, most of the times, this physician is the only health care professional working at the rural communities [17]. Therefore, the recently graduated physician must have the minimum medical competencies and confidence (translated into an adequate self-perception of the knowledge) to practice them. However, only two previous studies [18, 19] have assessed the competencies in this population. These studies were conducted in small samples, did not use a standardized list of competencies such as the Tuning Project (which prevents comparison with international publications), and did not assess the factors associated with having these competencies (which is necessary tailor interventions).

Given the need of information regarding this subject, we conducted the present study to describe the self-perception of competencies in clinical practice using Tuning Project dimensions and evaluated their associated factors among recently graduated physicians from Lima, Peru.

## 2 Methods

### 2.1 Study design and population

We carried out a cross-sectional study in physicians that had graduated less than one year ago from any of the medical schools located in Lima (Peru). Participants were recruited in the “VI SERUMS National Convention”, which was held in April 2017 and organized by the Peruvian Medical College. This event aimed to provide information and advice regarding the SERUMS to physicians who were starting the service in the following weeks. All medical schools in the city of Lima were invited to participate in this convention through their corresponding representatives.

The SERUMS is a rural social service that takes place in the first level of care and is a requirement for physicians to be able to work for the Peruvian government and to get into medical residency in Peru. Thus, most Peruvian physicians start SERUMS right after finishing their undergraduate studies [17].

The study population consisted of all recently graduated physicians who attended the event and who agreed to participate in the study. Those who had incomplete data for the variables of interest and those who did not pursue undergraduate studies in a Peruvian university were excluded from the analysis.

### 2.2 Procedures

Each physician was approached by trained pollsters when registering for the “VI SERUMS National Convention”. Pollster gave them a brief explanation about the study, invited them to participate, and handled them an informed consent form. Those who agreed to participate and signed the informed consent received a self-administered questionnaire and were explained that they could take as much time as needed to respond in the conference room. Two researchers tabulated all the questionnaires independently in an *ad hoc* database. Then, a third researcher compared the databases and inconsistencies were resolved by revising the questionnaires again.

### 2.3 Questionnaire

The questionnaire used for this study had four sections: 1) sociodemographic and academic data of the participant, 2) self-perceived competencies for general medicine, 3) self-perceived competencies for obstetric care, and 4) self-perceived competencies for mental health diagnosis and treatment. For this article, the first two sections were evaluated. The questionnaire was reviewed by experts and recently graduated physicians that did not attend the “VI SERUMS National Convention” who gave suggestions to make the questions clearer.

The section of self-perceived competencies for general medicine was constructed based on the adapted Tuning Project's dimensions for Latin America [20]. The “Tuning Project”, founded in 2000 by the European Commission, made a consensus (with the participation of graduates, employers, academics and students) with the purpose of establishing a list of basic competences for different careers (including medicine) and, therefore, standardize higher education programs in Europe [2, 5, 6]. Subsequently, the Tuning Project was contextualized in Latin America, with the participation of 19 universities in fifteen countries [4, 7].

The Tuning Project has eleven general dimensions:

1) **carry out an ambulatory consultation with a patient;**
2) **provide immediate care of medical emergencies;**
3) **carry out practical procedures**;
4) **apply the principles, skills, and knowledge of evidence-based medicine (EBM);**
5) assess clinical presentations, order investigations, make differential diagnoses, and negotiate a management plan;
6) communicate effectively in a medical context;
7) apply ethical and legal principles in medical practice;
8) prescribe drugs;
9) assess the psychological and social aspects of a patient's illness;
10) use information and information technology effectively in a medical context;
11) apply scientific principles, method, and knowledge to medical practice and research; and
12) work effectively in a health care system and engage with population health issues.

In the “self-perceived competences for general medicine” section, we included all the items of the first four dimensions (consultation, emergencies, procedures, and EBM), since we considered those to be the most important ones for recently graduated physicians.

### 2.4 Main variable: self-perception of basic medical skills

We assessed all 28 items of the selected dimensions: carry out an ambulatory consultation with a patient (6 items), provide immediate care of medical emergencies (6 items), carry out practical procedures (13 items), and apply the principles, skills, and knowledge of evidence-based medicine (3 items).

To assess each item, we used a Likert scale, as previous studies on the matter [8]. Each item was evaluated with the question “Do you consider that your level of competence in the following item is ...?”, scoring each of the possible answers on a Likert scale: “non-existent” (1 point), “insufficient” (2 points), “sufficient” (3 points), “good” (4 points), “very good” (5 points), and “excellent” (6 points).

### 2.5 Other variables

In addition, the following variables were studied: age, sex, the university where the participant completed his/her undergraduate studies (USMP: Universidad de San Martín de Porres, UPCH: Universidad Peruana Cayetano Heredia, UPC: Universidad Peruana de Ciencias Aplicadas, UNFV: Universidad Nacional Federico Villareal, UNMSM: Universidad Nacional Mayor de San Marcos, URP: Universidad Ricardo Palma, UPSJB: Universidad Privada San Juan Bautista, UCSUR: Universidad Científica del Sur), having done extracurricular externships for at least two months at any time during undergraduate studies (yes or no), and the hospital in which the participant did his/her internship (internship is done during the last year of medical education, and it could be done at hospitals of the Ministry of Health [MINSA], Social Security [EsSalud], and others [arm-forces and private institutions]).

### 2.6 Data analysis

For the descriptive analysis, we used absolute and relative frequencies (for the categorical variables), as well as measures of central tendency and dispersion (for the quantitative variables).

Linear regression models with bootstrap methods were used to calculate the coefficients (β) and their respective 95% confidence intervals (95% CI) for the associations between three covariates (the university in which the participant completed his/her undergraduate studies, having done extracurricular externships, and the hospital in which the participant did his/her internship) and the mean self-perception scores obtained from the four dimensions studied. All analyses were performed using the Stata v.14.0 software.

### 2.7 Ethical aspects

This study was approved by the Institutional Committee of Ethics in Research of the *Hospital Nacional Docente Madre-Niño - HONADOMANI* (RCEI-40). Participation was voluntary, and data confidentiality was maintained.

## 3 Results

A total of 693 physicians attended the “VI SERUMS National Convention” in 2017, from which 520 physicians accepted to participate in the study, of whom 95 were excluded (three for not having completed their undergraduate studies in Peru, 31 for having completed their undergraduate studies more than a year ago, and 61 for not answering any of the questions about self-perceived competencies). Finally, we analyzed data from 425 recently graduated physicians of the all of the universities in Lima (8 universities), which represent 31.1% (425/1368) of all physicians who graduated in 2016 from medical schools located in Lima [21].

Of these 425 participants, 227 (54.9%) were between 22 and 25 years old, 237 (55.8%) were women, 312 (73.6%) had completed their undergraduate studies in a private university, 130 (30.7%) had done extracurricular externships for at least two months, and 276 (65.7%) had completed their internship rotation at a MINSA hospital (Table 1).

**Table 1:** Characteristics of the study participants (n = 425).

Variables	N (%)
Age (n = 413)	
22–25 years	227 (54.9)
26 years	68 (16.5)
More than 26 years	118 (28.6)
Sex (n = 425)	
Male	188 (44.2)
Female	237 (55.8)
University (n = 425)	
USMP	116 (27.3)
UPCH	21 (4.9)
UPC	12 (27.3)
UNFV	34 (27.3)
UNMSM	63 (27.3)
URP	55 (12.9)
UPSJB	40 (9.4)
UCSUR	49 (11.5)
Others	35 (8.2)
Funding of the university where the participant completed undergraduate studies (n = 424)	
Public	112 (26.4)
Private	312 (73.6)
Doing an extracurricular externship for at least two months at anytime during undergraduate studies (n = 423)	
No	293 (69.3)
Yes	130 (30.7)
Hospital in which the participant did the internship (n = 420)∗	
MINSA	276 (65.7)
Hospital Nacional Dos de Mayo	36 (13.0)
Hospital Nacional Arzobispo Loayza	37 (13.4)
Hospital Nacional Hipólito Unanue	29 (10.5)
Others	174 (63.0)
EsSalud	71 (16.9)
Hospital Nacional Guillermo Almenara Irigoyen	17 (23.9)
Hospital Edgardo Rebagliati Martins	11 (15.5)
Hospital de Emergencias Grau	10 (14.1)
Others	33 (46.5)
*O*tros (sanidades and private)	73 (17.4)

MINSA: Peruvian Ministry of Health, EsSalud: Peruvian Social Security.

∗ For this variable, those who did their internship in more than one institution were excluded.

Concerning self-perception of physicians about their competencies, the average score for the dimensions evaluated was, in descending order: 4.49 for carrying out a patient consultation with a patient; 4.13 for carrying out practical procedures; 4.12 for providing immediate care of medical emergencies; and 4.04 for applying the principles, skills, and knowledge of evidence-based medicine.

The percentage of physicians who perceived that their competence was adequate (good, very good or excellent) was greater for the following items: measure blood pressure (96.2%), take a history (93.4%), suture wounds (92.7%), and carry out the physical examination (92.2%); and lower for the items: perform blood transfusions (33.6%), perform basic respiratory function tests (42.4%), provide advanced life support (48.5%), administer intravenous therapy and use infusion devices (51.8%), and perform and interpret electrocardiograms (53.4%) (Table 2).

**Table 2:** Self-perceived competencies of recently graduated physicians in Peru.

Competencies (Question: “Do you consider that your level of competence in the following areas is ...?”)	% of those who answered G, VG, or E∗	% of those who answered VG or E∗	Average score (from 1 to 6 points)
Dimension 1: Carry out an ambulatory consultation with a patient			
Take a history	93.4	62.4	4.67
Carry out physical examination	92.2	52.7	4.52
Provide reassurance and support	90.6	61.2	4.65
Provide explanation and advice	91.1	58.8	4.58
Make clinical judgements and decisions	88.9	43.1	4.36
Assess the patient's mental state	78.4	39.3	4.18
Average score			4.49
Dimension 2: Provide immediate care of medical emergencies			
Recognize and assess acute medical emergencies	86.6	46.1	4.37
Provide basic first aid	84.0	47.1	4.38
Provide basic life support and cardiopulmonary resuscitation	83.1	46.1	4.37
Treat acute medical emergencies	82.1	38.1	4.23
Provide trauma care according to current national guidelines	62.3	20.9	3.92
Provide advanced life support	48.5	13.9	3.44
Average score			4.12
Dimension 3: Carry out practical procedures			
Measure blood pressure	96.2	83.1	5.23
Suture wounds	92.7	65.9	4.83
Administer subcutaneous and intramuscular injections	84.5	59.3	4.64
Bladder catheterisation	79.1	49.6	4.39
Administer oxygen	75.3	45.2	4.31
Move and handle patients	74.1	40.5	4.20
Perform and interpret urinalysis	74.6	44.0	4.24
Venepuncture	73.6	45.2	4.27
Cannulation of veins	65.4	40.2	4.06
Perform and interpret electrocardiography	53.4	22.6	3.65
Administer intravenous therapy and use infusion devices	51.8	24.2	3.57
Perform basic respiratory function tests	42.4	14.8	3.27
Perform blood transfusions	33.6	13.6	3.05
Average score			4.13
Dimension 4: Apply the principles, skills and knowledge of evidence-based medicine			
Define and carry out appropriate literature search	73.6	39.3	4.17
Critically appraise published medical literature	67.8	32.0	4.02
Apply evidence to practice	65.9	29.2	3.93
Average score			4.04

∗ G: good, VG: very good, E: Excellent.

Regarding the factors associated with the average score obtained in each dimension, it was found that UPCH physicians had higher average scores in all dimensions, UPC physicians in two dimensions (“carry out practical procedures” and “apply the principles, skills, and knowledge of evidence-based medicine”), and UPSJB physicians only in the dimension of “carry out practical procedures”. On the other hand, only USMP physicians had a lower average score in “carrying out practical procedures” and “applying the principles, skills, and knowledge of evidence-based medicine.”

Additionally, we found that those who had done extracurricular externships for at least two months at any time during undergraduate studies obtained a higher average score in the dimensions of carrying out an ambulatory consultation with a patient and practical procedures and that those who had done internships at EsSalud hospitals had a higher average score than those who did them at MINSA hospitals, for “carrying out practical procedures” and “apply the principles, skills, and knowledge of evidence-based medicine” (Table 3).

**Table 3:** Factors associated with the average score obtained for self-perceived competencies in each dimension.

Dimension	Carry out an ambulatory consultation with a patient	Provide immediate care of medical emergencies	Carry out practical procedures	Apply the principles, skills and knowledge of evidence-based medicine
	β (95% CI)	β (95% CI)	β (95% CI)	β (95% CI)
University where the participant completed his/her undergraduate studies∗:				
USMP	-0.08 (-0.24 to 0.07)	-0.09 (-0.27 to 0.09)	**-0.20 (-0.38 to -0.03)**	**-0.21 (-0.41 to -0.02)**
UPCH	**0.63 (0.35 to 0.91)**	**0.60 (0.27 to 0.94)**	**0.48 (0.13 to 0.84)**	**1.05 (0.72 to 1.38)**
UPC	0.26 (-0.07 to 0.60)	0.32 (-0.06 to 0.70)	**0.40 (0.002 to 0.79)**	**0.76 (0.26 to 1.26)**
UNFV	-0.27 (-0.56 to 0.02)	-0.28 (-0.58 to 0.01)	-0.11 (-0.45 to 0.23)	-0.34 (-0.69 to 0.01)
UNMSM	0.02 (-0.17 to 0.21)	-0.04 (-0.37 to 0.29)	-0.12 (-0.34 to 0.10)	-0.11 (-0.40 to 0.17)
URP	-0.04 (-0.23 to 0.14)	0.01 (-0.21 to 0.24)	-0.03 (-0.24 to 0.18)	-0.08 (-0.29 to 0.14)
UPSJB	-0.01 (-0.19 to 0.17)	0.14 (-0.08 to 0.36)	**0.23 (0.02 to 0.44)**	0.05 (-0.19 to 0.29)
UCSUR	0.02 (-0.17 to 0.22)	-0.14 (-0.39 to 0.11)	0.06 (-0.17 to 0.30)	0.12 (-0.18 to 0.42)
Others	0.02 (-0.17 to 0.22)	0.10 (-0.16 to 0.36)	0.12 (-0.10 to 0.33)	0.10 (-0.16 to 0.36)
Did an extracurricular externship for at least two months at anytime during undergraduate studies∗∗:				
No	Ref	Ref	Ref	Ref
Yes	**0.18 (0.05 to 0.31)**	0.12 (-0.05 to 0.28)	**0.16 (0.01 to 0.32)**	0.10 (-0.10 to 0.29)
Hospital in which the participant did his/her internship∗∗:				
MINSA	Ref	Ref	Ref	Ref
EsSalud	0.18 (-0.003 to 0.37)	0.29 (-0.03 to 0.61)	**0.26 (0.03 to 0.49)**	**0.31 (0.05 to 0.57)**
Other	0.15 (-0.03 to 0.33)	0.16 (-0.04 to 0.35)	0.16 (-0.03 to 0.34)	0.18 (-0.06 to 0.42)

USMP: Universidad de San Martín de Porres, UPCH: Universidad Peruana Cayetano Heredia, UPC: Universidad Peruana de Ciencias Aplicadas, UNFV: Universidad Nacional Federico Villareal, UNMSM: Universidad Nacional Mayor de San Marcos, URP: Universidad Ricardo Palma, UPSJB: Universidad Privada San Juan Bautista, UCSUR: Universidad Científica del Sur.

MINSA: Peruvian Ministry of Health, EsSalud: Peruvian Social Security.

β coefficient; CI confident interval.

In bold: statistically significant associations.

∗ Each university was compared with the others (for example, the coefficient for USMP had as reference the rest of the physicians who did not study at USMP). Also the estimates were adjusted for variables externship and hospital.

∗∗ Regression multiple model that included the variables university, externship and hospital.

## 4 Discussion

### 4.1 Descriptive results

A survey performed in 2014 in Portugal in 1591 recently graduated physicians from seven medical schools aimed to evaluate the self-perception of the competencies proposed by the Tuning Project [8]. This study is very similar to ours. The self-perception average score in our study was higher for the “carrying out a consultation with a patient” dimension and lower for the “applying the principles, skills, and knowledge of evidence-based medicine” dimension. Conversely, the Portugal study found that, for the dimensions addressed by our study, the physicians reported a higher score for “applying the principles, skills, and knowledge of evidence-based medicine”, followed by “carrying out a consultation with a patient” [8]. This suggests that our population may have a disadvantage regarding evidence-based medicine competencies.

Competencies in evidence-based medicine are closely related to specific courses and training, in addition to the examples given by professors in the clinical setting [22, 23]. Thus, a possible explanation of the low self-perception of these competencies in our population is the lack of participation in evidence-based medicine courses, research centers, scientific societies of students, or rotations in hospitals where evidence-based medicine is deeply rooted in the clinical practice.

Within the “providing immediate care of medical emergencies” dimension, we found that items with a lower proportion of physicians self-perceived as competent included “providing advanced life support” and “providing trauma care according to current national guidelines”. This is in line with the results in the Portugal study [8]. Also, one study conducted in Zambia among physicians who had completed their internships found low perceived skills to provide advanced life support [24].

A possible explanation is that when severely traumatized patients or patients needing advanced life support go to hospitals, residents or specialists may be the ones who take care of these patients, giving little opportunity to students. In Peru, physicians can acquire or strengthen these competencies through specialized courses after undergraduate studies. However, the lack of these competencies among recently graduated physicians is worrisome, since most of these physicians are going lead care in rural services as soon as they finish their undergraduate studies [17].

In our study, the general procedures with the highest proportion of physicians who self-perceived as competent included: measuring blood pressure (96.2%), suturing wounds (92.7%), and administering subcutaneous and intramuscular injections (84.5%). Conversely, one study conducted in Zambia found that 37.5% and 14.3% felt very competent for administering intramuscular injections and suturing wounds, respectively [24], while in Portugal, the mean competence score (which ranged from 0 to 5) was 2.61 and 2.64 for self-perceived competence for suturing wounds and measuring blood pressure, respectively [8].

The general procedures with the lowest proportion of physicians who felt competent in our study included: performing blood transfusions (33.6%), performing basic respiratory function tests (42.4%), and administering intravenous therapy, and use infusion devices (51.8%). Similar to the findings in Portugal [8], the mean competence scores (which ranged from 0 to 5) for performing blood transfusions (0.82), administering intravenous therapy (1.47), and performing respiratory function tests (1.77) were the lowest. This may be because these activities are usually carried out by nurses, and medical schools do not emphasize its learning.

### 4.2 Associated factors

The higher self-perception in all dimensions of UPCH graduates in comparison with other universities could be related to the fact that this university has active clinical simulation centers [25], where theoretical knowledge is strengthened by practising in real situations [26]. It may also be associated with specific courses or with greater participation in research since this university is among those with the highest scientific output in Peru [27]. On the other hand, UPC graduates have a higher self-perception than other universities in the dimensions of “carry out practical procedures” and “apply the principles, skills, and knowledge of EBM”. UPC also have a curricular externship, case-based evaluations, and active clinical simulation centers [28, 29], but its first class of graduate physicians was in 2013 [30], so it is expectable that their medical curriculum is still under improvement.

Regarding the difference found between EsSalud and MINSA hospitals, it is possible that in some hospitals the academic aspect of the possibilities of performing procedures with patients is greater. However, it should be noted that eligibility to do an internship at EsSalud is achieved through a national examination among those who wish to apply, thus, students who are admitted to the internship program at EsSalud may have already on average more self-perceived skills than those of MINSA. However, in our study, we found a higher self-perception score in those physicians who carried out their internship in an EsSalud's hospital only in two dimensions (“carry out practical procedures” and “apply the principles, skills, and knowledge of EBM”). Despite this previous preparation of students who apply to the EsSalud internship have, it is necessary to also consider some characteristics of the hospital that could impact the development of medical competencies such as the administrative burden for the student [31] or the logistic (number of cases, number of interns, and residents) of the hospital, which reduces the number of cases in which students are involved and decreases individualized learning. Subsequent studies should explore these variables and their implications for the development of medical competencies.

We found an association between having done extracurricular externships and having a higher score in the competencies of carrying out an ambulatory consultation with a patient and practical procedures, which suggests that externships could be a useful intervention to improve procedural skills. However, it is also possible that students with greater motivation (and, therefore, better skills) are those who actively seek to do an externship. Thus, the direction of this association cannot be established, and it should be better studied.

### 4.3 Implications and recommendations

The shortcomings found in this study must be taken into consideration by Peruvian universities, which should ensure that all their physicians graduate with optimal competencies in general medicine [2]. Universities should consider a methodology to education according to the approach of competencies based on results, this being the current scene in medical training, that performed a holistic view of education based on a definition of the competencies as a combination of knowledge, skills, and attitudes [32].

Although, in Peru, level the theoretical knowledge acquired by physicians during undergraduate studies is evaluated through the national examination of medicine (ENAM, by its acronym in Spanish) and its results are taken into account for licensing medical schools [27], it is also interesting to evaluate the practical competencies or structured clinical examination using minimum competencies checklists (like “Objective Structured Clinical Examination” type tests), or at least students' self-perception of these, to have a wider perspective of the Peruvian medical education [24, 33].

The fact that the scores of self-perception of competencies differed across medical schools suggests that there may be differences in the medical education process. Thus, it is important to assess the differences in education contents and methodologies, and make efforts to ensure that medical students acquire a set of basic competencies needed for their labor as physicians and especially for the SERUMS.

### 4.4 Limitations

Some limitations of this study should be considered: 1) Although the ideal way to evaluate medical competencies is by observing these practices in real scenarios, we have opted to evaluate the self-perception of competencies as a proxy. Thus, some physicians may tend to over-estimate or under-estimate their real competences. However, there is a correlation between self-perceived and actual competences [24, 34], and self-perceived competence regarding certain items also evaluates the physician's motivation to perform such items (since those who do not feel competent will tend to avoid doing it) [35, 36]. Also, some studies have shown that self-perception is related to real knowledge [37]. Moreover, self-perceived competencies are important as self-perception of young physicians reflect the subject's motivation and can influence their attitudes towards performing a test, and so influence care decisions [38, 39]. 2) We analyzed data of the third part of physicians who graduated during 2016 from medical schools located in Lima, specifically those who attended the “VI SERUMS National Convention”. Physicians who did not attend to such convention may have some different characteristics, such as a lower rate of planning to perform the SERUMS, so the extrapolation should be performed carefully [40] 3) Our response rate was around 75%, which may bias the results in such a way that those who felt not competent in the evaluated dimensions may have chosen not to participate in the study. This would overestimate the perception of competencies found in our study. 4) Participants were physicians who planned to underwent the SERUMS, so we are probably loosing information from those who have decided to follow medical residency abroad, who may have higher self-perception of certain competencies. 5) Being an exploratory analysis, the influence of several potential confounders was not collected, so future studies are needed to confirm our results.

## 5 Conclusion

We evaluated the self-perception of competencies of 442 physicians. The average self-perception score was higher for the “carrying out a consultation with a patient” dimension and lower for the “applying the principles, skills, and knowledge of evidence-based medicine” dimension. The factors associated with this score were: having completed their undergraduate studies in certain universities, having done an externship for at least two months during undergraduate studies, and having done an internship at EsSalud (instead of MINSA) hospitals.

## Declarations

### Author contribution statement

W. Nieto-Gutierrez, J. Zafra-Tanaka, A. Taype-Rondan: Conceived and designed the experiments; Performed the experiments; Analyzed and interpreted the data; Contributed reagents, materials, analysis tools or data; Wrote the paper.

K.Pacheco-Barrios: Contributed reagents, materials, analysis tools or data; Wrote the paper.

### Funding statement

This work was supported by The Sociedad Científica Médico Estudiantil Peruana, Perú (SOCIMEP).

### Declaration of interests statement

The authors declare the following conflict of interests: The authors have completed undergraduate and postgraduate studies at Universidad de San Martín de Porres, Universidad Nacional Mayor de San Marcos and Universidad Peruana Cayetano Heredia.

### Additional information

No additional information is available for this paper.

## Appendix A Supplementary data

Heliyon.dta Heliyon.dta

## References

[1] Rizo C.A., Jadad A.R., Enkin M. (2002). What's a good doctor and how do you make one? Doctors should be good companions for people. Br. Med. J..

[2] Cumming A., Ross M. (2007). The Tuning Project for Medicine-learning outcomes for undergraduate medical education in Europe. Med. Teach..

[3] Tuning América Latina [Web Page] (2019). Tuning: 2004-2008. Proyecto Tuning América Latina.

[4] Tuning América Latina [Web Page] (2019). Tuning: 2011-2013. Medicina.

[5] Tuning Academy [Web Page] (2019). Tuning: 2009-2013. Medicine.

[6] Tuning educational structures in Europa [Web page] (2007). Tuning Educational Structures in Europe: Medicine in: Tuning Project.

[7] Hanne C. (2013). El proyecto Tuning latinoamericano: la experiencia del área de medicina. Rev. Hosp. Clin. Univ. Chile.

[8] Grilo Diogo P., Barbosa J., Ferreira M.A. (2015). A pilot Tuning Project-based national study on recently graduated medical students' self-assessment of competences-the TEST study. BMC Med. Educ..

[9] Monrouxe L.V., Grundy L., Mann M., John Z., Panagoulas E., Bullock A. (2017). How prepared are UK medical graduates for practice? A rapid review of the literature 2009–2014. BMJ Open.

[10] Svirko E., Lambert T., Goldacre M.J. (2014). Gender, ethnicity and graduate status, and junior doctors' self-reported preparedness for clinical practice: national questionnaire surveys. J. R. Soc. Med..

[11] Goldacre M.J., Taylor K., Lambert T.W. (2010). Views of junior doctors about whether their medical school prepared them well for work: questionnaire surveys. BMC Med. Educ..

[12] Katowa-Mukwato P., Andrews B., Maimbolwa M., Lakhi S., Michelo C., Mulla Y. (2014). Medical students' clerkship experiences and self-perceived competence in clinical skills. Afr. J. Health Prof. Edu..

[13] Huang C.C., Chan C.Y., Wu C.L., Chen Y.L., Yang H.W., Huang C.C. (2010). Assessment of clinical competence of medical students using the objective structured clinical examination: first 2 years' experience in Taipei Veterans General Hospital. J. Chin. Med. Assoc. : J. Chin. Med. Assoc..

[14] Galli A., Gregorio MJd (2006). Competencias adquiridas en la carrera de Medicina: comparación entre egresados de dos universidades, una pública y otra privada. Educ. Méd..

[15] Ortiz Moreira L.E., Gajardo Navarrete L.J. (2014). Propuesta de competencias genéricas para el perfil de egreso del médico cirujano de la Universidad de Concepción, Chile. Educ. Méd. Super..

[16] Villegas-Álvarez F., Polaco-Castillo A.J., González-Zamora J.F. (2007). García-Pineda AM, Madrid-Zavala MR. Competencias médico-quirúrgicas. Autopercepción en médicos recién egresados de la licenciatura. Cirugía Cir..

[17] Mayta-Tristán P., Poterico J.A., Galán-Rodas E., Raa-Ortiz D. (2014). El requisito obligatorio del servicio social en salud del Perú: discriminatorio e inconstitucional. Rev. Peru. Med. Exp. Salud Pública.

[18] Segovia E.V., Huayna L.S., Lezama C.C., Holguín E.M., Tristán P.M. (2013). Concordancia entre las habilidades, conocimientos y labores de médicos peruanos recién egresados con respecto a su propia percepción de las características de un buen médico. Rev. Med. Risaralda.

[19] Taype-Rondán Á., Inga-Berrospi F., Casiano Celestino R., Bastidas F. (2015). Percepción de médicos recién egresados sobre las habilidades clínicas adquiridas durante el pregrado en Lima, Perú. Rev. Med. Chile.

[20] Tuning P., Tuning América Latina (2007). Reflexiones y perspectivas de la educación superior en America Latina.

[21] Zafra-Tanaka J.H., Hurtado-Villanueva M.E., Saenz-Naranjo MdP., Taype-Rondan A. (2018). Self-perceived competence in early diagnosis of cervical cancer among recently graduated physicians from Lima, Peru. PloS One.

[22] Ma X., Xu B., Liu Q., Zhang Y., Xiong H., Li Y. (2014). Effectiveness of evidence-based medicine training for undergraduate students at a Chinese Military Medical University: a self-controlled trial. BMC Med. Educ..

[23] Alahdab F., Firwana B., Hasan R., Sonbol M.B., Fares M., Alnahhas I. (2012). Undergraduate medical students' perceptions, attitudes, and competencies in evidence-based medicine (EBM), and their understanding of EBM reality in Syria. BMC Res. Notes.

[24] Katowa-Mukwato P., Banda S. (2016). Self-perceived versus objectively measured competence in performing clinical practical procedures by final year medical students. Int. J. Med. Educ..

[25] Universidad Peruana Cayetano Heredia [Web Page] (2019). UPCH: Lima. Simulación Clínica.

[26] McGaghie W.C., Issenberg S.B., Cohen E.R., Barsuk J.H., Wayne D.B. (2011). Does simulation-based medical education with deliberate practice yield better results than traditional clinical education? A meta-analytic comparative review of the evidence. Acad. Med. : J. Assoc. Am. Med. Coll..

[27] Mayta-Tristán P., Toro-Huamanchumo C.J., Alhuay-Quispe J., Pacheco-Mendoza J. (2019). Producción científica y licenciamiento de escuelas de medicina en el Perú. Rev. Peru. Med. Exp. Salud Pública.

[28] Universidad Peruana de Ciencias Aplicadas [Web Page] (2009). UPC: Lima. UPC inaugura Centro de Simulación Clínica.

[29] Universidad Peruana de Ciencias Aplicadas [Web Page] (2020). UPC: Lima. Malla Curricular Medicina Humana.

[30] Universidad Peruana de Ciencias Aplicadas [Web Page] (2013). UPC: Lima. La primera promoción de la Escuela de Medicina de la UPC se ubica entre las tres primeras del país.

[31] Nieto-Gutierrez W., Taype-Rondan A., Bastidas F., Casiano-Celestino R., Inga-Berrospi F. (2016). Percepción de médicos recién egresados sobre el internado médico en Lima, Perú 2014. Acta Méd. Peru..

[32] Leung W.-C. (2002). Competency based medical training: review. BMJ.

[33] Martínez-González A., Sánchez-Mendiola M., Méndez-Ramírez I., Trejo-Mejía J.A. (2016). Grado de competencia clínica de siete generaciones de estudiantes al término del internado médico de pregrado. Gac. Med. Mex..

[34] Davis D.A., Mazmanian P.E., Fordis M., Van Harrison R., Thorpe K.E., Perrier L. (2006). Accuracy of physician self-assessment compared with observed measures of competence: a systematic review. Jama.

[35] Albert Bandura, Freeman wH. (1997). Self-efficacy: the Exercise of Control.

[36] Galli N. (1978). How self-perceived knowledge, actual knowledge and interest in drugs are related. J. Drug Educ..

[37] Kaduszkiewicz H., Wiese B., van den Bussche H. (2008). Self-reported competence, attitude and approach of physicians towards patients with dementia in ambulatory care: results of a postal survey. BMC Health Serv. Res..

[38] Lai N.M., Teng C.L. (2011). Self-perceived competence correlates poorly with objectively measured competence in evidence based medicine among medical students. BMC Med. Educ..

[39] Kaduszkiewicz H., Wiese B., van den Bussche H. (2008). Self-reported competence, attitude and approach of physicians towards patients with dementia in ambulatory care: results of a postal survey. BMC Health Serv. Res..

[40] Ministerio de Salud (2018). Evaluación de competencias a los profesionales médicos, obstetras y enfermeros del Servicio Rural y Urbano Marginal en Salud SERUMS. Salud-Investigación.

